# Analysis of Cytotoxicity of Selected Asteraceae Plant Extracts in Real Time, Their Antioxidant Properties and Polyphenolic Profile

**DOI:** 10.3390/molecules25235517

**Published:** 2020-11-25

**Authors:** Patrycja Sowa, Dana Marcinčáková, Michał Miłek, Ewelina Sidor, Jaroslav Legáth, Małgorzata Dżugan

**Affiliations:** 1 Department of Bioenergetics, Food Analysis and Microbiology, Institute of Food Technology and Nutrition, University of Rzeszów, Zelwerowicza 4, 35-601 Rzeszów, Poland; 2 Department of Chemistry and Food Toxicology, Institute of Food Technology and Nutrition, University of Rzeszów, Ćwiklińskiej 1a, 35-601 Rzeszów, Poland; mmilek@ur.edu.pl (M.M.); ewelina.sidor@poczta.onet.pl (E.S.); mdzugan@ur.edu.pl (M.D.); 3 Department of Pharmacology and Toxicology, University of Veterinary Medicine and Pharmacy in Košice, Komenského 73, 041 81 Košice, Slovakia; dana.marcincakova@uvlf.sk (D.M.); jaroslav.legath@uvlf.sk (J.L.); 4 Department of Biotechnology and Bioinformatics, Faculty of Chemistry, Rzeszów University of Technology, Powstańców Warszawy 6, 35-959 Rzeszów, Poland

**Keywords:** *Achillea millefolium*, adherence, antioxidant activity, cell line, metabolic activity, polyphenols, *Solidago gigantea*, *Tanacetum vulgare*, xCELLigence system

## Abstract

Plants from Asteraceae family are widely used for their therapeutic effects in the treatment of gastrointestinal diseases, but the consequences of excessive intake still need to be studied. The aims of this study were the evaluation of cytotoxicity, measurement of antioxidant properties and determination of polyphenolic profile of *Tanacetum vulgare* L. (tansy), *Achillea millefolium* L. (yarrow) and *Solidago gigantea* Ait. (goldenrod) ethanolic extracts. The cytotoxicity of extracts was monitored by xCELLigence system in real time by using porcine intestinal epithelial cell line (IPEC-1) and by measurement of changes in metabolic activity ((3-(4,5-dimethylthiazol-2-yl)-5-(3-carboxymethoxyphenyl)-2-(4-sulfophenyl)-2H-tetrazolium) (MTS) assay). The antioxidant properties were measured by spectrophotometric methods and polyphenolic profiles were determined by HPLC-DAD for 50% ethanol extracts (10% *w/v*). Strong cytotoxic effect was recorded for tansy and yarrow extracts (125–1000 µg/mL) by xCELLigence system and MTS assay. Conversely, a supportive effect on cell proliferation was recorded for goldenrod extracts (125 µg/mL) by the same methods (*p* < 0.001). The antioxidant activity was in good correlation with total polyphenolic content, and the highest value was recorded for goldenrod leaves, followed by tansy leaves, goldenrod flowers and yarrow leaf extracts. The goldenrod extracts were abundant with flavonoids, whereas phenolic acid derivatives predominated in the polyphenolic profile of tansy and yarrow.

## 1. Introduction

The assumption that natural medicines are safer than synthetic drugs has caused exceptional growth in human exposure to natural products such as plants, phytotherapeutic agents and phytopharmaceutical products [1]. Moreover, medicinal plants are increasingly explored by the food industry. They are used as sources of additives for functional foods and drinks because of their known health-promoting benefits. Special attention is currently focused on the content of phenolic compounds (such as phenolic acids and flavonoids), which exhibit antioxidant properties. These compounds have a strong ability to stabilise vegetable fats and protect organisms against the toxic effects of free radicals [2,3]. Unfortunately, some of them can be toxic to living organisms from a dose-dependent point of view. Therefore, it is essential to assess both potential toxicity and the anti-proliferative effect of such natural products before their application as food additives. 

Many medicinal plants are used in beekeeping, some as nectar flows, others as additives for enriched honey [4,5]. Goldenrod honey is considered to be particularly valuable in Poland, it is especially recommended to treat genitourinary system problems, its antioxidant activity is comparable with honeydew honey [6]. Beekeepers, when producing enriched honey and herbal honeys, often use herbal additives based only on their own sense and experience, without taking into account the possible negative effects that could be caused by excessive consumption. A particularly controversial plant is tansy (not admitted by the EU as a food additive due to their use in no alimentary fields [7]) and yarrow (containing bioactive component thujone, similar to tansy). Overdosing on these herbs may result in adverse effects, such as allergic reactions and dermatitis (yarrow) and abortifacient action (tansy) [8,9]. Toxic doses of these herbs have not yet been established for humans, only LD_50_ values for mice (oral administration) were reported: for *T. vulgare* 9.9 g/kg and for *A. millefolium* 1.5 g/kg of body mass [10,11].

The common tansy (*Tanacetum vulgare* L.) is a perennial herb belonging to the Asteraceae family, and is grown wild in the Northern hemisphere in Europe, Asia and North America [12]. *Tanacetum vulgare* has been cultivated in gardens and used as a spice in human diets [13]. Tansy has been used for treating rheumatism, ulcers, digestive disorders, fevers, colds and as a remedy for internal parasites as natural medicine. Several studies have shown that this plant possesses antioxidant, antibacterial, anthelmintic, anti-inflammatory, antihypertensive, emmenagogue, antispasmodic and diuretic activity [10,12,14,15]. However, the essential oil of tansy contains thujone, a toxic monoterpene which can cause convulsions as well as liver and brain damage. Hence the maximum content of this compound in foodstuffs and beverages has been established by laws in several countries [16]. Despite the presence of thujone, the essential oil contains many beneficial compounds such as camphor, borneol, artemisia ketone, α-pinene, and carvone [17]. Moreover, *T. vulgare* is a rich source of sterols (stigmasterol, campesterol, cholesterol), non-volatile sesquiterpene lactones (tanacetine, parthenolide, tanachine), flavonoids (including luteolin, quercetin, apigenin and their glycosides), phenolic acids (chlorogenic, caffeic and dicaffeoylquinic acids), and others [14,18]. 

*Achillea millefolium* (known as yarrow) also belongs to the Asteraceae family. It is widely distributed throughout the temperate climates of the Northern hemisphere but is also found in Australia and New Zealand [19]. This perennial herb has been used in traditional medicine for centuries as a treatment for hepatobiliary disorders, gastrointestinal complaints, inflammation, wounds, rheumatism, hysteria, as well as internal and external bleeding [20]. It was reported that *A. millefolium* exhibits anxiolytic, anti-inflammatory, tonic, antimicrobial, antispasmodic and diaphoretic properties [21,22]. Additionally, it contains numerous classes of compounds, mainly volatile constituents of essential oil (chamazulene, cineol, camphor, azulene and others), steroids and triterpenes (sitosterol, stigmasterol, campesterol, α- and β-amyrin), and polyphenols (flavonoids: myricetin, quercetin, apigenin, luteolin, kaempferol derivatives and phenolic acids: e.g., chlorogenic, caffeic, p-coumaric and ferulic) [23,24]. 

Giant goldenrod (*Solidago gigantea* Aiton) is a perennial herb from Asteraceae family. It occurs naturally in North America but has become an invasive species in a large area of Europe, as well as on other continents [25]. It is one of the most efficient producers of nectar, thus, it is ideal for cultivation to enrich bee-pollinated crops [26]. *Solidago* species show a wide spectrum of health-promoting properties, exhibiting antioxidant, antimicrobial, spasmolytic, anti- inflammatory, anti-obesity, gastroprotective, and diuretic effects, which have been proven in many studies [27,28,29,30]. However, the research mainly focuses on only two goldenrod species: *S. canadensis* and *S. virgaurea*, whereas the biological activities of *S. gigantea* have been poorly investigated [25,31,32]. This latter species has been shown to contain some flavonoids, such as quercetin derivatives (hyperoside, rutoside, isoquercitrin, quercitrin) and kaempferol [33].

Plant extracts are widely used in many fields; therefore, evaluation of cytotoxicity seems to be key for determination of non-cytotoxic concentrations at which they can be safely used [34]. Gastrointestinal cells are directly exposed to the consumed substances. Moreover, phytotherapy has always been an important part in the therapy of gastrointestinal diseases. Many studies showed that most of the plants affecting the gastrointestinal tract belong to the Asteraceae family [35].

The aim of the study was to evaluate the cytotoxic antiproliferative effects of four selected herbal extracts on the non-transformed porcine intestinal epithelial cell line (IPEC-1). The biological effect of extracts was compared with their antioxidant activity and polyphenolic profile.

## 2. Results and Discussion

### 2.1. Cytotoxicity Assays

**Figure 1:**
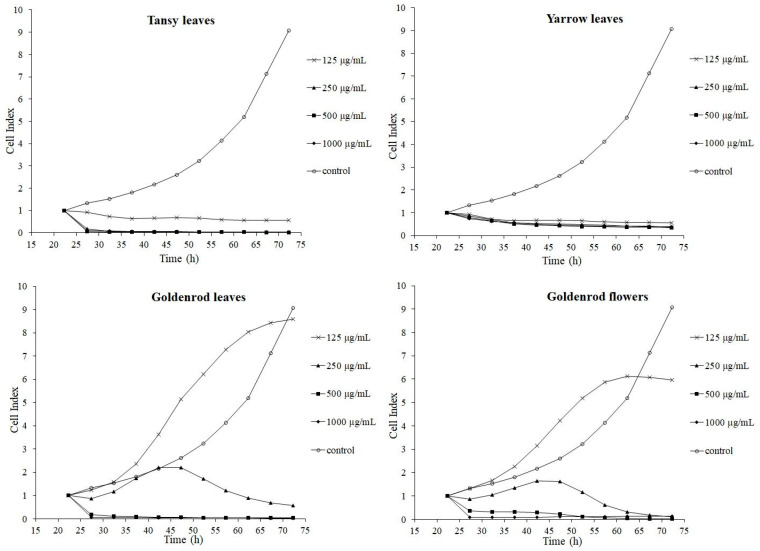
Cell index curves for porcine intestinal epithelial cell line (IPEC-1) cells incubated with tansy, yarrow, goldenrod leaves and goldenrod flowers.

In vitro cytotoxicity assays, which utilise cell lines, are widely used for evaluation of cytotoxicity of natural substances and chemicals, as well as for drug screening. They are rapid, inexpensive and do not require the use of animals. Moreover, they are able to test a large number of samples. In our experiment, Real-Time Cell Analyser xCELLigence system (RTCA; ACEA Bioscience, San Diego, CA, USA) was employed. This instrument has broad application prospects in various fields, such as drug screening, and development, toxicology analysis, pathology analysis, and herbal medicine. It plays an important role in the area of modern pharmaceutical evaluation and analysis [36]. It utilises patented microtiter plates (E-Plates^®^) which contain gold biosensors capable of monitoring the status of cells continuously and non-invasively in real time. The system gives information about behaviour of cells, their adherence, proliferation and change in morphology, and expresses them in a dimensionless unit: cell index (CI). The greater the CI, the greater the adhesion of cells [36,37]. Introducing the effector to cell culture induces the response of the target adherent cells and the corresponding activity (loss of adherence), which can be detected with great sensitivity and precision. Loss of adherence of adherent cells may lead to their death. The changes of CI were reported by generated real-time curves. The results are presented in Figure 1.

After the addition of tansy and yarrow extracts to cells, a strong cytotoxic effect was observed shortly thereafter, whereby CI significantly decreased to zero. This was observed at all tested concentrations, and we inferred that such action is associated with the presence of a large amount of terpene compounds in these plants [14,24]. 

After addition of lower concentrations of goldenrod leaves and flowers extracts (125 and 250 µg/mL), a positive effect on either cell adherence or proliferation was observed. On the other hand, higher concentrations (500 and 1000 µg/mL) caused a significant decrease in CI shortly after addition. This effect is frequently observed when plant extract contains polyphenols. Lower concentrations can support proliferation, while higher concentrations cause negative effects. Similar relationships have been previously observed in other experiments, for example: water-acetone extracts of dandelion (*Taraxacum officinale*) on the porcine epithelial renal cell line [37,38], anthocyanin-rich extracts of *Vaccinium angustifolium* on HepG2 cells [39] and *Oenothera paradoxa* extracts on Caco-2 cells [40]. Additional tests would be required to elucidate the mechanism of action of the tested extracts on cells.

The advantage of the xCELLigence system lies in its ability to monitor cell changes throughout the treatment with test substances and, based on CI changes, it can determine the duration of exposure and amount of extract at which the substance is beneficial to a living organism and when it becomes toxic and leads to changes in cellular morphology, proliferation or adherence. Based on CI values, it is possible to select a time suitable for subsequent end-point analyses, which will further determine the real state of the cells. To determine the true cell status at the end of the experiment, we used an end-point analysis MTS ((3-(4,5-dimethylthiazol-2-yl)-5-(3-carboxymethoxyphenyl)-2-(4-sulfophenyl)-2H-tetrazolium) assay. It evaluates changes in metabolic activity or viability of treated cells at the end of treatment (48 h). This protocol is increasingly used to evaluate the cytotoxicity of various plant extracts [41,42] and also for evaluating antiviral activity [43,44]. 

Results of this colorimetric test and CI values expressed as percentage of metabolic activity and percentage of adherence in comparison to control cells without treatment (100%) are summarised in Table 1.

The results of the MTS test show cytotoxic effect of the tested samples. Interestingly, it was observed that for the highest concentrations of extracts, the metabolic activity of cells was higher. However, it is important to note that a typical dose-dependent effect was not observed in all samples. The dependency of cytotoxicity on the concentration of the plant extracts has been tested by and proven via other studies [15,38,45], one of which utilised anthocyanin-rich berry extracts. Different observations recorded in our study may be caused by different compositions of extracts.

The contradictory results were observed for tansy and yarrow leaves. The metabolic activity increased while the adherence was lost. This effect could be caused by the higher amount of polyphenolic substances. In their investigation, Pagliacci et al. claimed that the polyphenolic flavonoid caused a decreased number of cells and cell cycle arrest of human breast cells while mitochondrial activity values were increased [46]. This statement is consistent with our findings. This observation may lead to underestimation of toxicity, thus more cytotoxicity assays are needed. For our experiment we decided to employ xCELLigence system for monitoring of cell response in real time. It is not a cytotoxic assay, but it provides important information about changes in cell proliferation and loss of adherence that may lead to death in adherent cells. Antiproliferative and cytotoxic effects of tested extracts are attributed to sesquiterpene lactones, volatile oils, flavonoids and phenolic acids. Such effects have also been reported by many other authors [15,47,48].

In our research, we analysed the chemical composition of the extracts that were prepared as required for testing with cell lines, so we did not identify thujone and other volatile compounds using HPLC-DAD method. Probably, these compounds can be crucial for further understanding the mechanisms of action at the cellular level.

### 2.2. Antioxidant Activity

The tested extracts were evaluated for the total content of phenolics and antioxidant capacity (Table 2). The highest content of polyphenols, 50.99 mg gallic acid equivalents per dry weight (GAE/g DW), was recorded for the extract of goldenrod leaves, followed by the tansy leaves and goldenrod flowers, while yarrow leaves were the poorest in terms of these compounds (*p* < 0.05). Similar content of polyphenols in tansy leaves was determined by Stojković et al. and Muresan et al. (46–50 mg GAE/g) [16,49]. Results obtained for yarrow leaves widely vary, values between 2.74 and 7.92 mg GAE/g DW have been reported by Georgieva et al. [23], and between 78 and 128 μg quercetin equivalents per gram by Keser et al. [21]. There are no data available for late goldenrod (*S. gigantea*) but the data available for other goldenrod species indicate a diverse species-dependent polyphenol content, ranging from 3.8 mg GAE/g for *S. canadensis* [27] up to 192 mg GAE/g for *S. graminifolia* [30]. The diversity of results is most likely due to the use of different solvents and extraction techniques. Antioxidant capacity of the tested extracts highly correlated with the content of phenols (r = 0.990 for total phenolic content (TPC) and ferric reducing ability of plasma (FRAP) and r = 0.981 for TPC and 2,2-diphenyl-1-picrylhydrazyl (DPPH)). This proves that polyphenolic compounds are by far the main antioxidants in this case. In addition, both methods used for determining antioxidant activity were closely correlated (r = 0.997). However, the correlation between antioxidant activity and results of MTS assay (after 48 h) and RTCA results (CI in 48 h) were insignificant (*p* > 0.05). The obtained results are in line with the literature data. Many authors describe the antioxidant properties of tansy, yarrow and goldenrod extracts. However, different methods are used and the results are expressed in different units, hence direct comparison of the results is often impossible. However, the high antioxidant potential of these plants was frequently emphasised [21,27,30,49]. 

### 2.3. Polyphenolic Profile

It is well known that plants belonging to the Asteraceae family contain mainly caffeoylquinic acid derivatives and flavonoids, especially apigenin, luteolin and quercetin derivatives [50,51,52,53,54]. The HPLC analysis in the present study confirmed these results (Table 3). Identified compounds were assigned to two main groups in order to determine which one predominated and which one played the greatest role in the biological activity of the herbs. The identification of individual compounds has been conducted by the analysis of UV–VIS spectra in comparison to the spectra of chemical standards and the literature data [55,56,57]. 

Chlorogenic acid, its isomers and derivatives were characterised by very similar spectra with λ_max_ at 326 nm, and shoulder at 296 nm. This group was the main fraction of phenolic compounds in tansy leaves as well as in yarrow, and chlorogenic acid was at an especially high amount in the former (37.64 mg/g—tansy). The level of these compounds in goldenrod flowers and leaves was twofold lower. Although caffeic acid and ferulic acid belong to hydroxycinnamic acids and have similar UV–VIS spectra to chlorogenic acid, they were analysed as a separate group. Caffeic acid was identified in all herbs, with obtained values ranging between 1.27 mg/g (goldenrod flowers) and 2.79 mg/g (goldenrod leaves), whereas ferulic acid occurred only in tansy and yarrow, at 14.22 and 0.27 mg/g content, respectively. Yarrow leaves contained both derivatives, ferulic and caffeic acid, but in a low amount. 

Flavonols derivatives hyperoside and rutin were identified based on retention times (comparison with analytical standards) and characteristic absorption maxima at 254 and 354 nm. Two clear bands, the first near λ = 250 nm and second 350 nm, are characteristic for flavone and/or flavonol-3-derivative, respectively. Generally, if the first band is more intensive than the second, it indicates the presence of a derivative of flavanol, but if the second band is more intensive, this indicates the presence of flavone [55]. Therefore, the peaks at the retention times 9.50 and 10.12 min were considered as flavanol derivatives. Two main flavone compounds were also identified: apigenin and luteolin. Apigenin has characteristic bands at 266 and 337 nm, whereas luteolin has characteristic bands at 253, 267 and 350 nm. The compound at 7.59 was considered to be glycoside derivative apigenin based on characteristic UV–VIS spectra at λ_max_ = 264 and 332 nm [50] and the last one as luteolin derivative (the same spectrum as aglycone). The identification of flavonol and flavone aglycones was much simpler than the identification of their glycosides. An important point to take into account is that tested yarrow extract was not found to contain any flavonol derivatives and only contained a small number of flavone derivatives (below 1 mg/g). On the other hand, high amounts of flavonol derivatives were determined in goldenrod leaves (46.17 mg/g) and flowers (26.68 mg/g). Rutin and one of the quercetin glycosides were only observed in the goldenrod extracts. The group of compounds that positively correlated with antioxidant activity were flavonol derivatives (r = 0.660 for DPPH and r = 0.717 for FRAP). Goldenrod leaf extract, which contained the highest content of these compounds, showed the highest antioxidant activity, while yarrow leaf extract in which flavonols were not identified showed the lowest. This may suggest that this group of polyphenols is mainly responsible for the antioxidant activity of the analysed extracts. The antioxidant activity of individual phenolic compounds depends on the number and positions of hydroxyl groups as well as the glycosylation of molecules [58]. 

The total phenolic content values, determined with Folin–Ciocalteu colorimetric method and HPLC-DAD technique, are positively correlated (r = 0.761). It should be noted that the Folin–Ciocalteu reagent is not specific for polyphenols. Its reduction can also be caused by other compounds present in plant extracts, e.g., amino acids, proteins, vitamins, carbohydrates, metal complexes, unsaturated fatty acids, which makes this method not entirely reliable for determining the total content of phenolic compounds. It has been postulated that it is better used to express the antioxidant activity of samples, however, it is still a popular method for determining the polyphenol content in plant extracts and food samples [59,60]. 

## 3. Materials and Methods 

### 3.1. Materials 

Plants: tansy (*Tanacetum vulgare* L.), yarrow (*Achillea millefolium* L.) and goldenrod (*Solidago gigantea* Ait.) were collected from a natural habitat (Rzeszów, Poland, 50°00′ N 22°01′ E) in September 2018 (minimum of ten plants per species). After botanical identification by authors based on morphological features of plants, the plant material was dried at room temperature, without exposure to sunlight. The voucher specimens were deposited in the Department archive (2018/09/TV1, 2018/09/AM1, 2018/09/SG1). Leaves of all species were analysed, and for goldenrod (which was collected at the flowering stage), flowers were also used. Chemicals used in TPC, DPPH and FRAP assays as well as analytical standards for chromatography were purchased form Sigma Aldrich (St. Louis, MO, USA).

### 3.2. Extracts Preparation 

The dry plant material was grounded in a laboratory mill (A11 IKA, Germany) to obtain a homogenous drug powder. Drug material (2 g) was extracted for 30 min with 20 mL of 50% (*v/v*) ethanol (Stanlab, Lublin, Poland) solution using an ultrasound-assisted method (U-504 Ultron, Moorpark, CA, USA). Subsequently, the extract was filtered through a paper filter. Ethanol was removed under vacuum using a rotary evaporator (Heidolph G3, Heidolph, Schwabach, Germany) at 40 °C. The extracts were prepared in triplicates. Obtained ethanol-free water extract was frozen at −80 °C and lyophilised for 24 h using FreeZone 2.5 lyophilizer (Labconco, Kansas City, MO, USA). Lyophilised extracts were diluted in sterile water, shortly before the experiment, to the final concentrations of 125, 250, 500 and 1000 µg/mL. 

### 3.3. Cell Cultivation

Intestinal Porcine Epithelial Cell line-1 (IPEC-1, CVCL_2245, Leibniz Institute, German Collection of Microorganisms and Cell Cultures, Braunschweig, Germany) were cultivated at a constant temperature of 37 °C, in 5% atmosphere containing CO_2_, in complete culture Dulbecco’s Modified Eagle’s Medium Nutrient Mixture F-12 (DMEM/F-12, Sigma Aldrich, Darmstadt, Germany), supplemented with 5% foetal bovine serum, 0.2% ITS (Insulin–Transferrin–Selenium, 500x concentrated, LONZA, Belgium), 0.05% Epidermal Growth Factor and 0.1% gentamicin (Sigma Aldrich, Darmstadt, Germany). Cells were passaged twice a week, using cells from the same passage for the experiment and regularly checked for the absence of mycoplasma contamination [61].

### 3.4. Real-Time Cell Analysis–xCELLigence System

For monitoring of cell response after addition of tested substances, xCELLigence system (Real-Time Cell Analyser—RTCA; Acea Biosciences Inc., San Diego, USA) was used. This system utilises a series of microelectrodes embedded on the bottom of microtiter plate wells to measure the number of cells attached, cell viability and the size and morphology of cells. Measurement is based on impedance changes caused by cell interaction with microelectrodes. Changes are expressed by a dimensionless unit cell index (CI) and recorded in curves. This method was described in various studies [37,38,62,63]. Initial cell seeding density was optimised using the xCELLigence system prior to the experiment. Firstly, 100 µl of cell medium DMEM was added into 16-well plates covered with gold microelectrodes (E-plates, Roche, Applied Science, Mannheim, Germany) for background measurements. Then, IPEC-1 cells (5 × 10^3^ cells per well in 50 µL of medium) were seeded into plates and inserted into plate station in an incubator kept inside a CO_2_ incubator at 37 °C with 5% CO_2_ and a humidified atmosphere. After 20 h, when cells were still within a log phase, the stock solutions of tested extracts were added to the cells (with cell medium at ratio 1:9) and the final concentrations were 125–1000 μg/mL. CI was recorded automatically by the RTCA system once per hour until the end of the experiment. Cells without the addition of extracts served as a control. 

Proliferative activity in percentual expression was calculated according to the following Equation (1):

% PA = CI_sample_ × 100/CI_control_ (1)

### 3.5. Cytotoxicity Assay 

To evaluate cytotoxic effects of tested extracts, MTS colorimetric assay (CellTiter 96^®^ Aqueous One Solution Cell Proliferation Assay, Promega, Madison, WI, USA) was used. This experiment was conducted simultaneously with RTCA and cells from the same passage were used. The cells were seeded into two 96-well plates (Greiner-Bio-One, Kremsmünster, Austria) in an amount of 8.6 × 10^3^ cells per well in 100 μL of medium. After 20 h, the same extract concentrations (125 to 1000 μg/mL) were added to cells and incubated for 48 h. Subsequently, 20 µL of MTS solution was added to each well and absorbance was measured at 490 nm after 4 h incubation using microplate reader (Synergy HT; Biotek, Winooski, VT, USA). The absorbance of the control cells was considered as 100%. All experiments were performed in triplicate.

### 3.6. Antioxidant Activity and Total Phenolic Compounds

DPPH Radical Scavenging Activity was measured based on procedure described by Dżugan et al. [6]. Briefly, 0.2 mL of plant extract was added to 1.8 mL of 0.1 mM DPPH (Sigma Aldrich Co., St. Louis, MO, USA) solution in methanol (Sigma Aldrich Co., USA), and left in dark for 30 min. Then, the absorbance was measured at λ = 517 nm using a UV–VIS Spectrometer (Biomate 3, Thermo, Madison, WI, USA). The obtained results were expressed as µmol Trolox (Sigma Aldrich Co., St. Louis, MO, USA)—Trolox equivalents (TE) equivalents per 1 g of dry weight of the extract based on prepared standard curve (5–60 nmol of Trolox solution in methanol).

FRAP Assay (ferric reducing antioxidant power) was also determined according to Dżugan et al. [6]. Briefly, 0.2 mL of plant extract was mixed with FRAP reagent (containing 2.5 mL of 10 mM TPTZ solution in 40 mM HCl, 2.5 mL of 20 mM FeCl_3_ and 25 mL of 0.3 M acetate buffer (pH 3.6)) and the absorbance of mixture was measured spectrophotometrically (UV–VIS Spectrometer Biomate 3, Thermo Sci., Madison, WI, USA) at 593 nm after 10 min incubation at 37 °C against blank. Calibration curve was prepared for Trolox (Sigma Aldrich Co., St. Louis, MO, USA) ethanol solution at the range 25–300 nmol/mL and the results were expressed as μmol of Trolox equivalents (TE) per 1 g of dry weight of the extract.

Total phenolic content (TPC) was also measured using procedure described by Dżugan et al. [6]. In summary, 0.2 mL of plant extract was mixed with 1 mL Folin–Ciocalteu reagent (diluted 10x) and 0.8 mL of 7.5% *w/v* sodium carbonate. After incubation at room temperature for 120 min, the absorbance was measured spectrophotometrically (Biomate 3, Thermo, Madison, WI, USA) at 760 nm against blank. TPC was calculated based on calibration curve at the range 25–250 μg/mL. Results were expressed as mg of gallic acid equivalents (GAE) per 1 g of dry weight of the extract. 

### 3.7. HPLC Analysis

Analyses were performed on a Gilson (Gilson’s Analytical-to-Semipreparative HPLC System, Gilson Inc., Middleton, WI, USA) system equipped with a binary gradient pump (Gilson 322), a column thermostat (Knauer, Berlin, Germany), autosampler with fraction collector (Liquid Handler GX-271) and a photodiode array detector (PDA, Gilson 172). The analytical column Nucleosil C-18 100-5 (250 × 4.6 mm) (Knauer, Berlin, Germany), thermostatted at 40 °C, was used for chromatographic separations. The mobile phase (1 mL/min) consisted of 0.1% (*v/v*) formic acid in water (phase A) and acetonitrile (Sigma Aldrich, St. Louis, MO, USA) (phase B). The samples were eluted by the following gradient: 10% B (1.5 min), 10–100% B (1.5–20 min), 100% B (20–25 min) and again 10% B to equilibrate column. The injected volume was 20 μL. The chromatograms were recorded at 254, 280, 320 and 360 nm. The phenolic compounds were identified and classified into the specific groups by their UV–VIS spectra, literature data and by comparison of their retention time values with values of standards. External standards were used for calibration, including: caffeic acid, ferulic acid, chlorogenic acid, rutin, hyperoside, apigenin, luteolin and quercetin. The identified peaks were quantified as equivalents of the following standards: chlorogenic acid isomers as chlorogenic acid, other hydroxycinnamic acid derivatives as cinnamic acid, flavonols derivatives as hyperoside equivalents, and flavone derivatives as apigenin equivalents, which were then expressed using calibration curves at concentrations ranging from 0.005 to 0.1 mg/mL (R^2^ ≤ 0.9998). The results were expressed as mg/g of dry weight of the extract (mg/g DW). Before the chromatographic analysis, extracts were filtered through a 0.22 µm syringe nylon filter (Merck Millipore, Darmstadt, Germany). 

### 3.8. Statistical Analysis 

Results were expressed as means ± standard deviation (SD; *n* = 3). The significant differences (*p* < 0.05) between the analysed herbal extracts based on the antioxidant activity and total phenolic compounds content were determined using one-way analysis (ANOVA), followed by Tukey’s multiple comparison test. Statistically significant influence of extracts on metabolic activity of the IPEC-1 cells was evaluated by one-way analysis of variance (ANOVA) with Dunnett’s test. The correlation between studied parameters was calculated using Pearson’s correlation test. Statistical analysis was performed using Statistica 13.1 software (StatSoft, Inc., Tulsa, OK, USA). 

## 4. Conclusions

The analysed extracts from selected wild plants of the Asteraceae family showed a high antioxidant potential and content of phenolic compounds: phenolic acids and flavonoids derivatives. Cytotoxicity studies using the IPEC-1 cell line indicate yarrow (*Achillea millefolium*) and tansy (*Tanacetum vulgare*) as cytotoxic plants, which clearly indicates that these plants can be dangerous and should be used carefully in human and animal nutrition. In the case of goldenrod (*Solidago gigantea*) leaves and flowers, the results are more promising; a properly selected dose can support the proliferation of intestinal epithelial cells and may potentially be used in protection of this tissue. However, the correlation between cytotoxicity and antioxidant properties was not found. Tansy must be used carefully due to undesired effects after higher doses application. On the other hand, use of goldenrod seems to be safer. The positive effects of plant extracts are tested often, but evaluation of their cytotoxicity is also necessary.

## Figures and Tables

**Table 1:** The effect of the extracts on adherence and metabolic activity of IPEC-1 cells after 48 h of incubation.

Concentration of Extracts (µg/mL)						
		125 µg/mL	250 µg/mL	500 µg/mL	1000 µg/mL	Control
Tansy leaves						
RTCA	CI	0.13 ± 0.01 ***	0.07 ± 0.01 ***	0.05 ± 0.01 ***	0.08 ± 0.02 ***	4.07 ± 0.74
	A (%)	3.28 ± 0.28 ***	1.64 ± 0.14 ***	1.31 ± 0.14 ***	1.88 ± 0.51 ***	100 ± 18.23
MTS	MA (%)	42.95 ± 1.54 ***	49.25 ± 0.00 ***	66.42 ± 2.45 ***	82.15 ± 4.05 ***	100 ± 0.00
Yarrow leaves						
RTCA	CI	1.15 ± 0.07 ***	0.72 ± 0.09 ***	0.59 ± 0.07 ***	0.64 ± 0.11 ***	4.07 ± 0.74
	A (%)	28.34 ± 1.64 ***	17.61 ± 2.09 ***	14.41 ± 1.60 ***	15.72 ± 2.70 ***	100 ± 18.23
MTS	MA (%)	46.03 ± 1.77 ***	50.45 ± 1.28 ***	59.51 ± 1.44 ***	76.47 ± 1.71 ***	100 ± 0.00
Goldenrod leaves						
RTCA	CI	9.18 ± 2.78 ***	3.74 ± 0.62	0.08 ± 0.01 ***	0.07 ± 0.01 ***	4.07 ± 0.74
	A (%)	225.55 ± 68.26 ***	91.81 ± 15.31	1.88 ± 0.28 ***	1.64 ± 0.14 ***	100 ± 18.23
MTS	MA (%)	61.22 ± 3.27 ***	49.59 ± 1.67 ***	57.32 ± 2.02 ***	76.81 ± 2.80 ***	100 ± 0.00
Goldenrod flowers						
RTCA	CI	8.60 ± 2.56 ***	2.42 ± 0.23	0.31 ± 0.10 ***	0.12 ± 0.03 ***	4.07 ± 0.74
	A (%)	211.38 ± 62.87 ***	59.46 ± 5.54 ***	7.70 ± 2.56 ***	3.03 ± 0.79 ***	100 ± 18.23
MTS	MA (%)	60.12 ± 4.53 ***	47.74 ± 2.53 ***	51.44 ± 1.20 ***	62.04 ± 0.66 ***	100 ± 0.00

RTCA—real-time cell analyser (xCELLigence system); MTS—(5-(3-carboxymethoxyphenyl)-2-(4,5-dimethyl-thiazoly)-3-(4-sulfophenyl) tetrazolium, inner salt assay; CI—cell index; A—adherence; MA—metabolic activity. Significantly different compared to the control (Dunnett’s test *** *p* < 0.001).

**Table 2:** Total phenolic content and antioxidant capacity of tested plant extracts.

Extract	Antioxidant Capacity		
	TPC (mg GAE/g)	DPPH Reduction (μmol TE/g)	FRAP (μmol TE/g)
Tansy leaves	41.35 ± 1.48 ^a^	257.17 ± 16.69 ^a^	243.21 ± 3.02 ^a^
Yarrow leaves	15.86 ± 2.73 ^d^	92.19 ± 4.92 ^c^	84.17 ± 6.04 ^d^
Goldenrod leaves	50.99 ± 1.63 ^c^	272.47 ± 5.68 ^a^	272.25 ± 19.94 ^c^
Goldenrod flowers	32.58 ± 0.94 ^b^	192.82 ± 4.11 ^b^	192.76 ± 5.59 ^b^

TPC–total phenolic content; GAE–gallic acid equivalents; DPPH–2,2-diphenyl-1-picrylhydrazyl; TE–trolox equivalents, FRAP–ferric reducing ability of plasma. Data as mean value ± standard deviation (SD; *n* = 3). Means sharing the same superscript letter (a–d, in a column) are not significantly different (Tukey’s honest significant difference test, *p* < 0.05).

**Table 3:** Content of phenolic compounds (mg/g) in tested plant extracts.

Compound (mg/g)	Retention Time (min)	Tansy Leaves	Yarrow Leaves	Goldenrod Leaves	Goldenrod Flowers
Chlorogenic acid der	3.27	n.d.	1.92 ± 0.05	1.24 ± 0.02	0.72 ± 0.01
	6.99	8.41 ± 0.11	1.95 ± 0.02	0.46 ± 0.02	1.27 ± 0.04
	8.80	n.d.	0.33 ± 0.01	n.d.	n.d.
	10.14	n.d.	0.26 ± 0.01	n.d.	n.d.
Chlorogenic acid	6.25	37.64 ± 0.24	23.22 ± 0.80	8.92 ± 0.50	11.08 ± 0.13
Caffeic acid	2.91	1.33 ± 0.12	1.58 ± 0.02	2.79 ± 0.01	1.27 ± 0.02
Caffeic acid der	2.58	n.d.	0.89 ± 0.02	n.d.	1.92 ± 0.01
Ferulic acid	4.50	14.22 ± 0.13	0.27 ± 0.01	n.d.	n.d.
Ferulic acid der	5.33	n.d.	0.21 ± 0.01	n.d.	n.d.
Unknown phenolic acid	2.20	4.92 ± 0.05	n.d.	n.d.	n.d.
**Hydroxycinnamic acid derivatives total**		**66.52 ± 27.32**	**28.74 ± 10.56**	**12.17 ± 4.69**	**15.54 ± 6.29**
Rutin	7.85	n.d.	n.d.	1.25 ± 0.03	0.43 ± 0.02
Hyperoside	8.31	6.67 ± 0.60	n.d.	4.40 ± 0.50	7.70 ± 0.09
Quercetin Glycoside der	9.50	1.63 ± 0.03	n.d.	5.98 ± 0.02	7.95 ± 0.09
	10.12	n.d.	n.d.	34.54 ± 0.10	10.60 ±0.03
Luteolin	13.91	1.17 ± 0.01	0.19 ± 0.01	<LOQ	n.d.
Luteolin der	13.49	0.85 ± 0.02	n.d.	n.d.	n.d.
Apigenin	15.70	0.78 ± 0.02	n.d.	n.d.	0.16 ± 0.01
Apigenin der	7.59	2.52 ± 0.03	0.47 ± 0.05	n.d.	n.d.
**Flavonoid derivatives total**		**13.62 ± 4.89**	**0.66 ± 0.20**	**46.17 ± 15.46**	**26.84 ± 4.37**
**Total polyphenols**		**80.14**	**29.40**	**58.34**	**48.12**

der—derivative, n.d.—not determined, <LOQ—compound presented in sample under limit of quantification. Data as mean value ± standard deviation (SD; *n* = 3). The mean values for the group of compounds were emphasized in bold.
